# *Music and Movement for Health: *Protocol for a pragmatic cluster-randomised feasibility pilot trial of an arts-based programme for the health and wellbeing of older adults

**DOI:** 10.12688/hrbopenres.13535.1

**Published:** 2022-05-26

**Authors:** Amanda M. Clifford, Orfhlaith Ni Bhriain, Steven Byrne, Pui-Sze Cheung, Quinette Louw, Liam Glynn, Hilary Moss, Desmond O'Neill, Catherine B. Woods, Ali Sheikhi, Rosemary Joan Gowran, Catherine Maher, Brendan Kennelly, Jon Salsberg, Lehana Thabane

**Affiliations:** 1 School Of Allied Health, Ageing Research Centre, Health Research Institute, University of Limerick, Limerick, V94T9PX, Ireland; 2 Irish World Academy of Music and Dance, University of Limerick, Limerick, V94DK18, Ireland; 3 Division of Physiotherapy, Department of Health and Rehabilitation Sciences, Stellenbosch University, Cape Town, South Africa; 4 School of Medicine, Health Research Institute, University of Limerick, Limerick, Ireland; 5 Health Research Institute, University of Limerick, Limerick, Ireland; 6 Centre for Ageing, Neuroscience and the Humanities, Trinity College Dublin, Dublin, Ireland; 7 Department of Physical Education and Sport Sciences, Physical Activity for Health Research Cluster, Health Research Institute, University of Limerick, Limerick, Ireland; 8 Assisting Living and Learning (ALL) Institute, Maynooth University, Maynooth, Ireland; 9 Rehabilitation Unit, Community Hospital of the Assumption, Thurles, Tipperary, Ireland; 10 Cairnes School of Business and Economics, National University of Ireland Galway, Galway, Ireland; 11 Department of Health Research Methods, Institute for Research in Ageing, McMaster University, Canada

**Keywords:** Music Therapy, Dance, Arts, Health, Wellness, Older Adults

## Abstract

**Background:** Arts-based health programmes (ABHP) can enhance the physical and psychosocial health and wellbeing of older people. However, the feasibility and usefulness of such programmes in Ireland are currently unknown. The primary aim of this study is to examine the feasibility of the study design, its application to a music and movement for health programme and associated costs. The secondary aim is to obtain preliminary effect estimates of an ABHP on health and wellbeing in older adults.

**Methods:** This study is a pragmatic cluster-randomised controlled feasibility trial. Community-dwelling adults, aged 65 years or older will be recruited in the mid-west region of Ireland via methods including social prescription, traditional and social media. The clusters, based on geographical region, will be block randomised to either the ABHP or control using 1:1 allocation ratio. The programme will comprise a 1.5-hour music and dance session each week for 12-weeks together with a 1-hour home-based music and movement programme for 12-weeks. A qualitative and quantitative process evaluation of the arts-based health programme will be performed.

**Outcomes:** Primary outcomes for feasibility include recruitment rates (the number of participants recruited per cluster per month); retention rate (the number of participants who complete measures at baseline and at follow up post intervention, and minimum average attendance. Secondary outcomes will include physical function, balance, physical activity, loneliness, social isolation, cognition, mood, as well as quality of life and cost.

**Conclusions:** If this pioneering study finds evidence to support feasibility and acceptability, a future larger-scale definitive trial will be conducted to examine the effectiveness of an arts-based health programme for older adults. This research aims to strengthen collaborative efforts to implement effective, sustainable and cost-effective programmes for older adults to support community connection, enhancing health and wellbeing, in turn reducing demands on the healthcare system.

**ISRCTN registration:** ISRCTN35313497 (18/02/2022).

## Introduction

Ireland has an ageing population which is increasing and has significant healthcare challenges ( DoH, 2016b). A large proportion (61%) of older adults have a chronic condition ( DoH, 2016b) and are not meeting the recommended physical activity guidelines ( Murtagh *et al.*, 2015). Additionally, the risk of falling increases with age, with one in three older people falling each year ( HSE, 2008), leading to a loss of confidence, and reduction in physical function and social interaction ( Yardley & Smith, 2002). Almost one-third of adults aged 50+ in Ireland report loneliness, which was found to be most prevalent among those over 75 years and those living alone ( Ward *et al.*, 2019). More recently, the consequences of the COVID-19 pandemic and associated measures led to increased levels of social isolation, loneliness and physical deconditioning ( Dahlberg, 2021; Krendl & Perry, 2021; Robb *et al.*, 2020). Loneliness and social isolation are associated with poor health outcomes comparable to the dangers of smoking and obesity ( Cacioppo *et al.*, 2015; Perissinotto *et al.*, 2012). The financial and health implications of chronic conditions, falls, low levels of physical activity and loneliness reflect the failure of national frameworks and strategies ( DoH, 2013; DoH, 2016a; DoH, 2018; Ward *et al.*, 2019) to implement preventative strategies effectively ( Frieden, 2010). Such findings reaffirm the need for pioneering projects that are community-based and utilise community resources.

Research demonstrates that older adults tend to prefer physical activity that develop social connections and promote fun as well as increase accessible skills with benefits to their daily lives ( Devereux-Fitzgerald *et al.*, 2016; O’Regan *et al.*, 2020). Compared to exercising alone, group exercises foster social cohesion and connectedness and can potentially lead to greater adherence in older adults ( Kattenstroth *et al.*, 2010; Rivera-Torres *et al.*, 2019). Recent research provides evidence to support arts and creative interventions in improving health; playing a critical role in health promotion; helping to prevent or delay cognitive and physical decline and frailty ( IPH, 2021; WHO, 2019). Occupation-focused, meaningful, group-based programmes such as dance and music are shown to enhance physical and mental health, wellbeing and belonging ( Shanahan *et al.*, 2017; Stewart & Irons, 2018). Dance is an art form involving physical exercises such as partnered multidirectional movements, weight shifting and single leg stepping. Systematic reviews have found that dance can improve physical health with positive effects on strength, endurance, functional fitness and some risk factors associated with falls including balance and mobility ( Fernández-Argüelles *et al.*, 2015; Hwang & Braun, 2015; Liu *et al.*, 2021). Currently the findings are mixed for gait outcomes ( Fernández-Argüelles *et al.*, 2015; Liu *et al.*, 2021). Additionally, positive effects have been reported for the effects of dance and dance movement therapy on depression, anxiety, quality of life, interpersonal, cognitive and psychomotor skills ( Koch *et al.*, 2019). However, limitations exist in the current evidence base including small sample sizes, diversity in dance prescription, variable outcomes, the absence of tailoring and participant involvement in the programme design. Music can have an important role in emotion, communication and social interaction in aging. Indeed, musical activities can support positive aging. Listening and singing to music have been associated with better emotional well-being, competence and reduction in social isolation ( Hays & Minichiello, 2005). A Cochrane review ( van der Steen *et al.*, 2018) of music-based activities for older people with dementia and a systematic review and meta-analysis ( Zhao *et al.*, 2016), identified that providing at least five sessions of a music-based therapeutic programme may reduce depressive symptoms and may improve emotional well-being, quality of life and reduce anxiety. The literature demonstrates that music and dance can contribute to preventative health and well-being strategies for older people. However, despite the evidence that demonstrates the health benefits of both music and dance for older adults, research examining the physical and emotional benefits as well as the feasibility of a music and dance programme for older adults in Ireland is lacking. Prior to a conducting a large-scale study to examine the effectiveness of music and dance for older adults, a pilot trial is necessary to determine the feasibility and acceptability of the study design and evidence-based, participant-informed arts-based programme.

Consequently, the primary aim of this study will be to examine the trial and intervention feasibility by assessing the recruitment and retention rates, fidelity and acceptability of a co-designed arts-based programme study processes. The secondary aim is to obtain preliminary information to describe the effect of the programme on function, mobility, social isolation, loneliness, physical activity, cognition, mood and quality of life measures in older adults living in the mid-west of Ireland. An embedded pilot health economic analysis will also be conducted in conjunction with the feasibility trial to analyse the health economic data that will be collected in the feasibility trial and inform the health economics section of any subsequent trial. Ethical approval was granted for the study by the University of Limerick, Faculty of Education and Health Sciences Research Ethics Committee (EHSREC Ref No: 2022_01_08_EHS; Date of Approval: 26/01/2022).

## Methods

**Figure 1.**
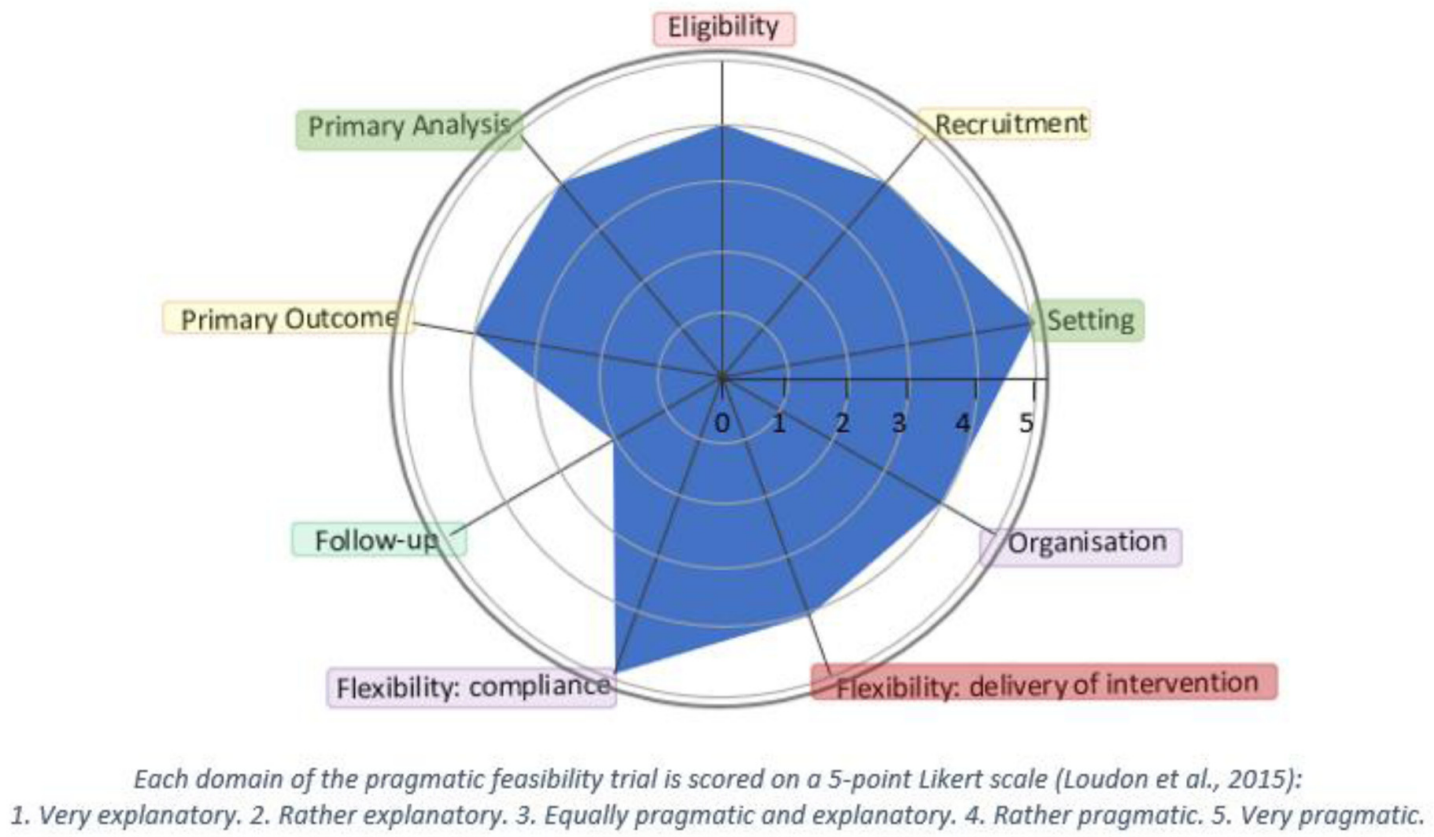
The Pragmatic-Explanatory Continuum Indicator Summary 2 (PRECIS-2) wheel-Music and Movement for Health ( Loudon *et al.*, 2015). This figure is an original figure produced by the authors for this article.

The *Music and Movement for Health* study is a pragmatic cluster-randomised, controlled feasibility trial with an embedded economic evaluation and process evaluation. The methodology was guided by the PRECIS-2 tool, which is illustrated in Figure 1 ( Loudon *et al.*, 2015). Reporting of the trial conforms to the SPIRIT checklist ( Chan *et al.*, 2013), the Consort extension to cluster-randomised trials checklist ( Campbell *et al.*, 2012), and the checklist of the CONSORT extension for feasibility and pilot trials checklist ( Thabane *et al.*, 2016; Thabane & Lancaster, 2019). Completed checklists and other study materials can be found in the *Extended data* and *Reporting guidelines* sections ( Clifford, 2022).

As evident in Figure 1, a broad consultative approach was used to ensure our eligibility criteria are appropriate, representative, and inclusive; and exclusion is aligned with usual care considerations. We will use a variety of recruitment strategies and envisage that most of the participants will be recruited via usual pathways, including social prescription by professionals as well as advocacy groups, newspapers and social media. The setting is both community and home based and not a controlled environment. Qualified dance teachers and music therapists will deliver the arts-based programme to participants randomised to receive the programme. The programme will be designed so that it can be integrated and implemented into the community setting. As such, the programme could easily occur in the community without the extensive use of resources. The *Music and Movement for Health* study contains key ingredients to ensure fidelity in content and delivery but sufficiently flexible to accommodate preferences, skill level and the needs and abilities of the various community cohorts. We are not introducing special measures to incentivise compliance. However, we will monitor participation using a log and include behavioural change techniques (BCTs) to optimise participation in the programme ( Kok *et al.*, 2016). Our primary outcomes are feasibility and acceptability, which are relevant to inform a future definitive trial and the secondary outcomes are relevant to the participants. Our analysis will include data from participants in both intervention and control groups regardless of their level of engagement during the intervention.

### Participants and recruitment

Two transparent expert consultation (TEC) workshops ( Higginson *et al.*, 2013) (EHSREC No: 2021_09_12_EHS) were held with health care practitioners (public health nurses, General Practitioners (GPs), community physiotherapists), music therapists, dance teachers and researchers to produce a stakeholder-informed specification on participant inclusion/exclusion criteria and recruitment pathways considering inclusivity and safety. As such, those who contributed to the TECs had extensive expertise and experience in rehabilitation, medical care or arts-based programmes (music/dance) for older people living in the community. This method is particularly useful when seeking to generate evidence-based recommendations for promoting the engagement of older people in physical and social activities ( Brighton *et al.*, 2018; Yardley *et al.*, 2007). The recruitment procedures were also informed by the *Move for Life* (MFL) programme ( O’Regan *et al.*, 2020).

### Patient and public involvement (PPI)

This study has embedded Patient and Public Involvement (PPI) in its overall approach and processes. As such, to ensure that the *Music and Movement for Health* project is informed by those who might engage with the programme now and into the future, a six-member PPI panel was established to support the research programme. This PPI panel has been instrumental in our processes thus far, with membership from: Age and Opportunity Ireland, Dance Limerick, dance teachers and older adult dancers. To date, PPI actions have included contribution to study design, outcome measures, intervention planning, programme logo, programme title, recruitment strategies and information posters/leaflets. It is anticipated that the future contributions of the PPI panel will include all aspects of research planning and delivery, attending progress meetings; interpreting results; the dissemination and communication of the research findings; and in the creation of a sustainable arts-based intervention. PPI meetings will be held online or face-to-face and members will be compensated for their participation. There are also flexible opportunities for involvement by e-mail, phone and post.

### Inclusion criteria

Community-dwelling older adults 65 years of age and older will be invited to participate in the study. Eligible participants will be able to walk three metres with or without an assistive device, have a basic level of English literacy or an identified person who can translate English to their first language and be able to hear and follow instructions.

### Exclusion criteria

To ensure participants are safe to exercise they will be excluded if they *subjectively* report health conditions that contraindicate their participation in exercise such as having had an acute stroke (within the past three months) or have an unstable neurological, cardiac or respiratory condition (or are on oxygen therapy). Participants will also be excluded if they do not satisfy the requirements of the Physical Activity Readiness Questionnaire (PARQ) ( Shephard, 2015).

### Recruitment

A purposive sampling strategy will be used to recruit older people for the 12-week *Music and Movement for Health* programme. For the 12-week programme, we plan to recruit individuals in the mid-west of Ireland from a minimum of six geographical regions across three counties (Limerick, Clare and Tipperary) in the mid-west of Ireland. Limerick city and county was reported to have the second highest level of total income per person in the State, after Dublin in 2014. However, Limerick also has more areas of deprivation and a lower labour force skillset profile compared to other cities; with the highest number of unemployment blackspots in which 35.7% of the workforce declared themselves unemployed. Limerick also has the lowest number of recreational facilities nationally. Recreational opportunities may also be more difficult to access in more marginalised or rural areas ( CSO, 2016). In 2016, approximately 65% of people in county Clare lived outside of larger towns (towns with more than 1,500 people) ( Clare County Council, 2018). Tipperary is one of the most populated rural counties in Ireland, with a strong network of vibrant and robust towns and villages with service centres provided at strategic locations throughout the county. There are many areas in Tipperary with an ageing population and over 25% of the population aged over 65 years live alone ( Tipperary County Council, 2021). For this study, participants will be recruited through social prescription (e.g., public health nurses, general practitioners, physiotherapists and occupational therapists), adverts in community areas (church bulletins, local sports clubs) and local newspapers, linking with volunteer organisations, primary care centres and community centres and word of mouth vis-à-vis the use of social media. Each centre will be selected for their ease of access for local community members, parking facilities, recreational facilities (tea/coffee availability), suitability for the programme (space and floor type) and ease of mobility within the building itself (flights of stairs etc.).

### Sample size

As our primary outcomes are feasibility it is desirable to keep the sample size small enough to allow close monitoring of participants and the programme while large enough to obtain sufficient data on our primary outcomes of feasibility. For feasibility reasons we need to ensure we have sufficient numbers in each group to generate a ‘group’ climate likely to maximise adherence. Hence if the group gets too small, it becomes unviable for participant engagement and sense of community. The minimum numbers required to sustain a community-based music and movement programme and maintain the required group dynamics is between 10–12 older adults per group; guided by practice expertise within the research team and previous feasibility studies ( Clifford *et al.*, 2021; O’Regan *et al.*, 2020). The primary feasibility outcomes will be evaluated by assessing recruitment rates (the rate of participants recruited to the study); retention rate (the number of participants who complete measures at baseline and at follow up post intervention) and minimum average attendance (number of participants who attended at least 65% of the intervention sessions). Hence, we aim to recruit a minimum of 72 participants in total (n=12 per cluster) allowing for 20% attrition (n=60; n=10 per cluster), we will be able to estimate a recruitment rate of 60% within a 95% confidence interval +/- 12.6%; a retention rate of 80% within a 95% confidence interval of +/- 10.3% and a minimum average attendance of 65% within a 95% confidence interval of +/- 12.3%. Every effort will be made to achieve a gender balance amongst the participants through our recruitment strategies, including recruitment sites and poster images.

### Randomisation

Pragmatic randomized controlled trials (PrCTs) are conducted to answer the important question of how an intervention works in real-world contexts in a heterogeneous real-world population. Thus, we plan to recruit individuals in the mid-west of Ireland from at least six geographical regions or clusters; the groups of study participants are the units of randomisation (clusters), and individuals within these groups are the units of analysis. The clusters are based on the geographical regions where the participants reside (two clusters in each county n=6 geographical regions, with n=2 additional reserve clusters sites). Using block randomisation, the clusters will be randomised to either the intervention or control (usual care) using 1:1 allocation ratio. Thus, there will be an intervention and a control group in each county. Given the wide geographical spread of these locations, it would be impractical to conduct an RCT by randomising participants individually to either the intervention or control arm and then find a central location to conduct the intervention. Additionally, this approach would not keep the community groups together. The cluster RCT design will ensure we keep community groups together. Research from a recent cluster-randomized controlled study, demonstrated that being ‘ *part of a local community group*’ can afford additional benefits, including an increased likelihood to remain active due to the friendships they had formed (OR=3.04, 95% CI=1.65–5.60, p=0.001), as a result of attending of the programme ( O'Regan *et al.*, 2021). In line with our pragmatic approach the study will have two phases for recruitment and two implementation blocks as the intervention can apply naturally to the group. Participants in the same group will receive the same intervention, thus approximating clinical practice, and producing generalisable findings.

### Concealed allocation

The trial statistician (AS) will be responsible for generating the random allocation sequence using R (R Core Team and the R Foundation for Statistical Computing) ( R-Project, 2022). The six clusters (plus two reserve clusters) were randomised into two trial arms, or blocks (intervention and control). Thus, each site was randomly assigned to one trial arm, making sure the equal number of clusters in each arm. In addition, two sites were randomly assigned into each arm as additional reserve sites. A member of the research team will be responsible for informing participants of their allocation and maintaining an undisclosed record of each cluster's allocation in line with recommendations ( Eldridge *et al.*, 2016). To minimise expectation bias, the personnel involved in recruitment (such as general practitioners, clinicians or local champion), assessment and analysis of the quantitative data will be blind to cluster allocation. Concealed allocation procedure will be followed, detailed and monitored. Only the relevant people will have access to the allocation data, which will be secured, and password protected.

### Music and Movement for Health Programme

The programme will be a community-based music and dance programme delivered by a dance teacher and music therapist over 12 weeks. The use of theory in intervention development is crucial to increase intervention effectiveness and facilitate behaviour change ( Aunger & Curtis, 2016; Fernandez *et al.*, 2019). As such, the programme and its mode of delivery have been designed following the Medical Research Council (MRC) guidance on developing complex interventions ( O'Cathain *et al.*, 2019) and guided by the Multi-level Leisure Mechanisms Framework ( Fancourt *et al.*, 2021). The intervention development process included: (1) reviewing published research evidence and examining existing protocols for facilitating music, singing and dance groups with older people in the community, (2) drawing on existing theories and techniques to facilitate participation and behavioural change including Social Cognitive Theory ( Bandura, 1998; Kelder *et al.*, 2015) and Self Determination Theory ( Deci & Ryan, 2004), as the research demonstrates the integration of multiple theories can effectively facilitate physical activity engagement with older adults and 3) involving stakeholders through PPI panel feedback and TEC stakeholder consultation workshops. In addition, the programme will include co-design with the participants to tailor the programme to each community group. The Multi-level Leisure Mechanisms Framework was used to support the design of a more holistic, theory driven, and cross-disciplinary intervention. The framework emphasises the key underlying processes including psychological, biological, social, behavioural, and health behaviour at play when promoting health ( Fancourt *et al.*, 2021), and highlights the potential mechanisms that could link the *Music and Movement for Health* project to wider health outcomes.

Each session over the course of the 12-week intervention will be structured to include a warm-up, music, singing and dance, creative response, followed by a cool-down a social tea and chat where participants will be encouraged to ask questions, provide feedback, and will be facilitated by Behavioural Change Techniques to incorporate music and dance into daily activity ( French *et al.*, 2014). This approach will be adopted as the health promotion research demonstrates that employing theory-based methods increases intervention effectiveness in changing behaviour ( Fisher *et al.*, 2002; Van Achterberg *et al.*, 2011). A playlist of participant’s personalised music will be developed in conjunction with the programme to facilitate participation in a home programme, whereby participants will be asked to perform the exercises to music. Home programme completion will be monitored using a self-report daily log. All participants will be advised to report any adverse effects and safety will be monitored during the sessions.

### Control group

The control group will be a true control usual care comparison group of participants. However, this group will be offered the chance to participate in the arts-based programme at the end of data collection if they desire. Participants will be asked to complete a falls log and continue with any usual concomitant care. However, people will be advised not to take up any new physical activity that includes dancing and/or music, which could have a confounding effect on the outcomes of the trial.

**Figure 2.**
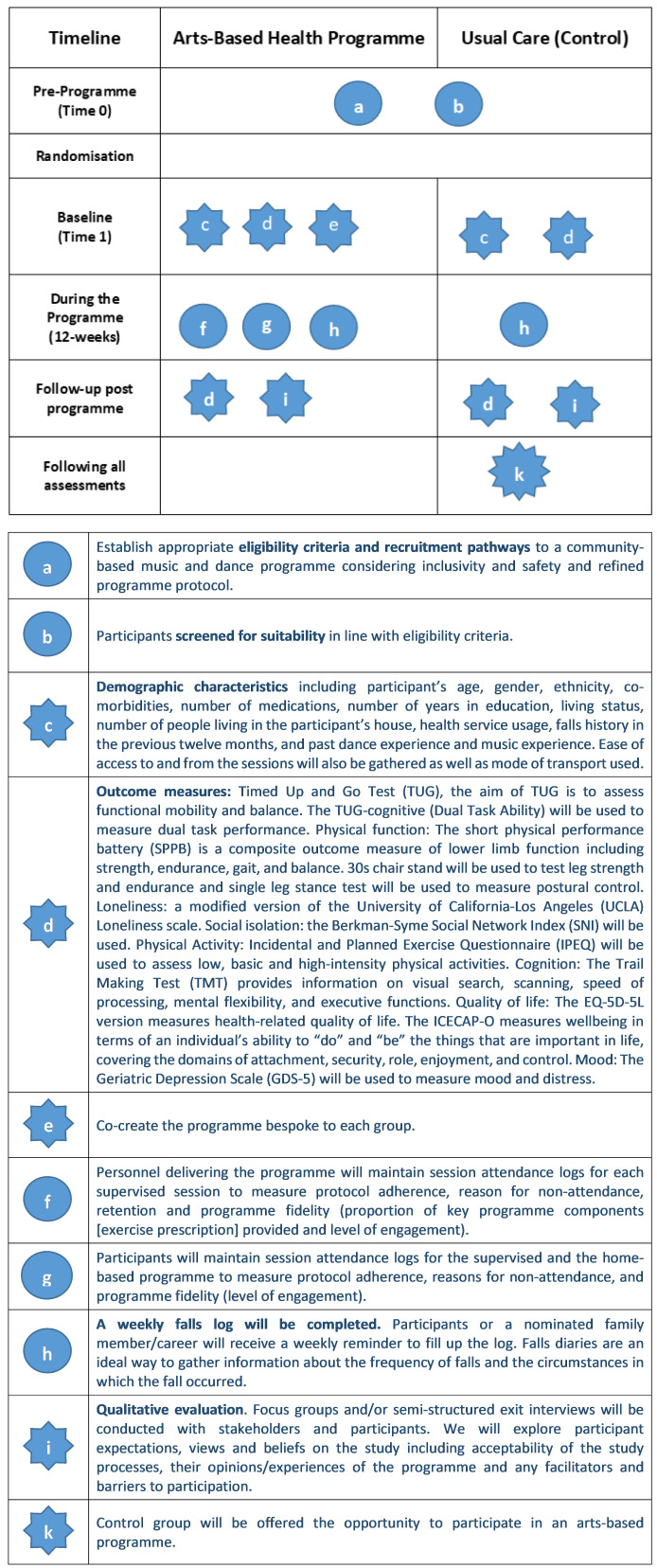
Graphical method for depicting randomised trials of complex programmes ( Perera *et al.*, 2007).

Individuals will discontinue in the programme if they develop any severe adverse events during the study which may/may not be related to the programme and would affect a participant’s ability to participate in the programme safely (such as chest pain or an injurious fall). In the case of a participant developing a severe adverse event they would require further medical investigation and GP clearance to continue their participation. Participants can also choose to discontinue with the programme at any time for any reason. A summary of the study design can be found in Figure 2.

### Assessment procedures

Participants will be screened and assessed at baseline and at follow-up after the 12-week programme by blinded assessors. At baseline, demographic characteristics including participant’s age, gender, ethnicity, number of years in education, living status, number of people living in the participant’s house, co-morbidities, number of medications, health service usage, falls history in the previous twelve months, and past dance experience and music experience will be recorded. Eligible participants that consent to participate will be advised not to change their exercise habits and to inform the research team of any changes made to their medical or exercise regime during the study. Feasibility of the programme will be assessed quantitatively by reporting:

- Recruitment rates: This will be assessed by reporting the recruitment rate (as the number of participants recruited and randomised per centre per month and the number of people enrolled over the number of people who expressed an interest in taking part). Refusal rates will also be documented where provided.
- Retention: The proportion of individuals who after enrolment completed the baseline and follow-up assessments. We will record numbers who completed baseline and assessment at 12-week follow-up and attendance at programme. We will calculate the minimum outcome assessment target, which is set at a retention rate of at least 80% of recruited participants with valid baseline and 12-week primary outcome data i.e., less than 20% attrition at outcome assessments at baseline and follow-up.
- Attendance at supervised sessions: We will record the percentage of participants that attend the supervised sessions (adherence). Minimum average attendance target will be determined as the minimum average attendance by recruited participants set as 65%.
- Participation in home exercise programme: Using a self-complete calendar we will record how many participants (and how often) they engage in the home exercise programme.
- Safety will be assessed through self-report of direct or unrelated adverse events (e.g., falls or pain during the programme period). Falls: A falls diary will be completed. Participants or a nominated family member/career will receive a weekly reminder to complete the log. Completing a falls diary encourages recall, identifies the key circumstances surrounding falls and their content focuses the programme. The falls diary will include: Date and time of fall; location of fall; what caused the fall; type of injury sustained; and action taken. The falls diary is more accurate than retrospective recall over varying time periods.

The variability, consistency, response rates and data completeness for each of the secondary outcome measures will be determined. The secondary outcomes of the study have been used in previous studies with older adults, have undergone psychometric testing and include:

### Physical performance measures

- Timed Up and Go Test (TUG): The aim of the TUG is to assess functional mobility and balance while performing a sequence of functional tasks. It is a simple, valid, reliable and cost-effective measure to re-produce in a clinical setting as it requires the participant to stand up from a chair, walk three meters, turn around and walk back to sit on the chair again. TUG scores are associated with future falls in older adults, TUG time ≥12.6 seconds (adjusted for age, gender, comorbidities, medications, and past history of two falls) was significantly associated with future falls ( Kojima *et al.*, 2015).
- TUG-Cognitive (Dual Task Ability): The TUG-Cognitive will be used to measure dual task performance during the TUG. The time to complete the test is used to score dual task performance with faster times indicating better performance. This test has high criterion validity and very good test-retest reliability ( Hofheinz & Schusterschitz, 2010).
- The 30 Second Sit to Stand Test, also known as the 30 second chair stand test (30CST), assesses the lower extremity strength and endurance in community-dwelling older adults. The test provides a reasonably reliable and valid indicator of lower body strength in generally active older adults ( Rikli & Jones, 1999).
- Single Leg Stance Test is a measure of static postural control. This test requires participants to stand on one leg with their hands on their hips, unaided. The time is tracked for 30 seconds. Higher scores indicate better postural control. Scores of under five seconds are suggestive of greater postural deficiencies ( Jonsson *et al.,* 2004). Excellent reliability has been found for this test (ICC 0.99) ( Springer *et al.,* 2007).
- Short physical performance battery (SPPB): The SPPB measures lower limb function including strength, endurance, gait, and balance through three components, walking speed, chair stands, and standing balance. The SPPB is able to predict mortality and functional mobility limitations in older adults ( Treacy & Hassett, 2018). Scores range from 0 to 4 (0= inability to complete the task; 4= highest level of function) for each task of SPPB with the sum of these three tests (0–12) reflecting the complete measurement of physical function.

### Self-report physical activity

- Incidental and planned exercise questionnaire (IPEQ): The IPEQ is a self-report questionnaire for assessing low, basic and high-intensity physical activities. It is suitable for use in ageing research including falls prevention trials. The IPEQ covers the frequency and duration of planned activities and incidental physical activities in older adults. A total activity score is obtained by multiplying frequency scores and duration scores. The IPEQ can discriminate between physical activity levels across groups that differ by fall risk factors. It has been shown to have strong test-retest reliability, validity, and internal consistency ( Delbaere *et al.*, 2010).

### Loneliness

- UCLA Loneliness Scale: In line with the *Irish LongituDinal Study on Ageing* (TILDA), we will measure loneliness using a modified version of the University of California-Los Angeles (UCLA) Loneliness scale ( Russell, 1996). This scale will provide a self-report measure of emotional loneliness and thus the individual’s subjective satisfaction with the quality of their social relationships, which is considered the psychological embodiment of social isolation.

### Social Isolation

- Berkman-Syme Social Network Index (SNI): Again in line with TILDA, we will measure social isolation using the Berkman-Syme Social Network Index (SNI) ( Berkman & Syme, 1979). This index categorises individuals into four levels of social connection using a 0-4 composite scale, whereby, socially isolated is scored at 0-1; moderately isolated at 2; moderately integrated at 3; and socially integrated at 4. The four types of social connections are: (1) marital status; (2) sociability (number and frequency of contacts with children, relatives and friends); (3) active church group membership; and; (4) membership in other community organisations.

### Cognition

- The Trail Making Test (TMT) is used to measure cognitive functioning. The TMT involves attention, visual screening ability, processing speed, mental flexibility, and executive functions. Part A of the TMT requires an individual to draw a continuous line to connect 25 encircled numbers that are randomly distributed on a page. Part B of the TMT requires individuals to connect 24 circles with numbers and letters, switching between numbers and letters in consecutive order. The score on each part represents the amount of time required to complete the task. The TMT has shown to be a reliable and valid outcome measure in healthy older adults ( Cangoz *et al.*, 2009).

### Quality of Life and wellbeing and mood

- The EQ-5D-5L measures quality of life using a scale from 1 to 5 across five domains (mobility, self-care, usual activities, pain, and anxiety/depression) and a visual analogue scale from 0 to 100 to determine subjective physical health. Compared to the EQ-5D-3L, research demonstrates that the EQ-5D-5L has a reduced ceiling effect and increased reliability as well as an improved ability to discriminate between different levels of health ( Janssen *et al.*, 2013).
- ICEpop CAPability measure for Older people (ICECAP-O): ICECAP-O was developed for use in the economic evaluation of health and social care programmes. It measures wellbeing based on an individual’s capability to ‘do’ and ‘be’ the things that are important to them. ICECAP-O covers five domains that were found to be important to older people: attachment, security, role, enjoyment and control ( van Leeuwen *et al.*, 2015).
- The 5-item Geriatric Depression Scale (GDS-5) will be used to measure mood. The GDS-5 was chosen to evaluate the effect of the music and movement programme on the mood of the participants. GDS-5 is a tool for measuring depression in older adults. The short version GDS-5 has sufficient sensitivity (97%) and good reliability. The questionnaire is composed of fives items to which it is necessary to answer ‘yes’ or ‘no’. This form can be completed in approximately 1 to 3 minutes ( Hoyl *et al.*, 1999).

A pilot health economic analysis will be conducted in conjunction with the feasibility trial. The health economic evaluation will evaluate the costs and outcomes of the programme jointly so as to understand the economic effectiveness of the *Music and Movement for Health* programme. For the cost-utility analysis, Quality Adjusted Life-Years (QALYs) will be estimated using data collected via the EQ-5D-5L survey instrument at baseline and over the follow-up period. Missing data will be analysed to explore the validity of the data collection tools and the timing of their application. An incremental analysis will be conducted to calculate the mean differential in costs and QALYs between the control group and the programme group. The incremental cost-effectiveness ratio (ICER) will be calculated and analysed. Parametric and probabilistic analyses will be employed to explore uncertainty. The evidence generated from the pilot study will go to inform the design and conduct of a full economic evaluation to assess the cost-effectiveness of the developed programme.

### Process evaluation

*Music and Movement for Health* will include a mixed-methods process evaluation. For the quantitative process evaluation, personnel delivering the programme and participants will maintain session attendance logs for each session to measure protocol adherence, reason for non-attendance, retention and programme fidelity (proportion of key programme components [exercise prescription] provided and level of engagement). To assess the suitability of the eligibility criteria and success of the recruitment strategies, we will evaluate if referred participants represent our target population, the reasons for refusal, success of the various recruitment strategies and if sufficient recruitment rates were achieved. For the qualitative process evaluation, semi-structured interviews and focus groups will be used with stakeholders and participants depending on their availability and preference. We will explore participant expectations, views and beliefs on the study including acceptability of the study processes, their opinions and experiences of the programme and any facilitators and barriers to participation. Research recommends a combination of interviews and focus groups as it is pragmatic and can enrich the data. The focus groups will facilitate development and discussion of a variety of views and ideas through participant interaction in a comfortable, safe and supportive environment. The interviews will enable an in-depth understanding of the individual's experience and enables the exploration of personal perspectives and interconnectivity in personal perspectives. The focus groups and interviews will be arranged for a convenient time and location and will be audio-recorded and transcribed verbatim. Similarly, a qualitative evaluation of the programme will be conducted with the people involved in recruitment, referral and delivery of the programme. We will explore their views on the study including facilitators and barriers to upscaling to a full programme. A summary of the study’s key objectives can be found in Table 1.

**Table 1.** Summary of the key study objectives, outcomes and analysis plans.

Aim	Objectives	Outcome	Criterion for Feasibility	Statistical Analysis
**Primary**	Assess feasibility of recruitment, retention, group size and data completion.	Recruitment and retention rates, missing data.	*Music and Movement for Health* is feasible. The rate of participants recruited to the study, retention rate (the number of participants who complete measures at baseline and at follow up post intervention, minimum average attendance (number of participants who attended at least 65% of the intervention sessions). We will calculate the minimum outcome assessment target, as a retention rate of at least 80% of recruited participants with valid baseline and 12-week primary outcome data.	To assess if we can recruit and retain sufficient eligible older adults to inform a future definitive trial. Descriptive statistics: mean and SD for continuous variables proportions for dichotomous variables.
	Assess safety of the programme.	Safety: adverse effects e.g., falls, a weekly falls log will be completed.	The programme is safe. There is no increase in direct adverse events due to the intervention (e.g., falls during the intervention period).	Safety assessed through self-report of direct or unrelated adverse events (e.g., falls or pain during the programme period).
	To obtain participants’ & stakeholder feedback and experiences of the study processes and programme.	Qualitative study feedback.	*Music and Movement for Health* is acceptable. The majority of participants report that the study processes including measures were feasible and acceptable.	Thematic analysis. Data regarding combined completion and acceptability of measures.
	Objective	Outcomes	*Hypothesis*	Statistical Analysis
**Secondary**	To explore the differences between the groups for Physical Function, Balance, Physical Activity, Loneliness, Social Isolation, Mood, and Quality of Life.	Results for Clinical outcomes: Physical Function, Balance, Physical Activity, Loneliness, Social Isolation, Mood, and Quality of Life.	The programme group will show improvement in scales.	Regression analysis
	To explore the differences between the groups for Physical Function, Balance, Physical Activity, Loneliness, Social Isolation, Mood, and Quality of Life.	Results for each outcome of interest.	We can determine the required sample size for a definitive trial.	ICC (intraclass correlation coefficient) will be used to calculate the design effect. Thus, the required sample size for a CRCT (cluster-randomised controlled trail) will be calculated by multiplying the required sample size for the equivalent ungrouped study with the DE.
	Pilot economic analysis to assess costs and inform a definitive trial.	A pilot economic analysis to inform the design and conduct of a full economic evaluation to assess the cost effectiveness of the programme developed.	The methods developed and implemented for the conduct of the pilot health economic analysis proved to be feasible and acceptable to study participants.	The criterion for success includes obtaining sufficient data on cost. Incremental analysis to calculate the mean differential in costs and QALYs. The incremental cost effectiveness ratio (ICER) will be calculated and analysed. Parametric and probabilistic analyses will be employed to explore uncertainty.

### Data analysis

The quantitative data collected for this research will be input into IBM SPSS Statistics software package (version 27). As this is a feasibility study, the analysis will focus on descriptive statistics and confidence interval estimation. Continuous variables will be reported as means, standard deviation and 95% confidence intervals, and categorical data as the number and percentages. A content analysis will be performed on any data from open-ended questions. Qualitative data collected for this research will be analysed according to Braun and Clarke's 6-step framework for doing inductive thematic analysis, including familiarisation and theme generation, searching, reviewing and defining ( Braun & Clarke, 2019). Investigator triangulation will support integrity in the data analysis process. Thus, sample transcripts will be independently coded by members of the research team to discuss and agree on the codes and interpretations. The research team will meet to discuss the dataset and develop consensus around coding, interpretations, patterns, ideas and themes. NVivo software (London, United Kingdom) will be used to code and analyse the data using built-in functionality. The themes will be reviewed and discussed by all members of the research team to generate a thematic 'map' of the analysis.

### Trial Governance and data management

This trial will be conducted in accordance with international standards of good practice in clinical trials (ICH GCP Good Clinical Practice). A Trial Management Committee (TMC), a Trial Steering Committee (TSC) and Independent Data Monitoring Committee (IDMC) will be convened to ensure adequate trial management, governance and safety monitoring for the trial. The Health Research Institute (HRI) Clinical Research Support Unit Limerick (CRSU Limerick) will provide support with document development, data quality, data storage, and archiving of data in line with good clinical practice (GCP) standards as well as FAIR (findable, accessible, interoperable, and reusable) principles for scientific data management and stewardship. An electronic database (CRFWEB) has been developed to facilitate data collection and management. CRFWEB is a secure cloud-based electronic data capture software which enables the researchers to collect, integrate, and manage data. CRFWEB will run regular data validation checks, provide a range of data audit and data trails, automatically raised queries and offer multiple layers of security. The independent data monitoring committee (IDMC), which includes an independent trial statistician, the CRSU manager, and an independent clinician, will monitor data emerging from the trial, in particular in relation to safety and efficacy, and make any recommendations as required to the Trial Steering Committee (TSC).

### Ethical approval

Ethical approval for this study has been granted by the Faculty of Education and Health Sciences Research Ethics Committee at the University of Limerick (EHSREC No: 2022_01_08_EHS). During the recruitment phase, potential participants will be provided with information about the study, where they will have the opportunity to read about the study and ask any questions about the study prior to providing written informed consent to participate. The information sheet will contain all relevant information, including the aim, nature of the study, procedures, confidentiality, possible risks, benefits, and freedom to withdraw without consequence in advance of deciding to participate. The research team will preserve the confidentiality of participants taking part in the study in line with GDPR guidelines.

### Plans for dissemination of the study outcome

Dissemination of the research findings will follow the World Health Organisation Research Dissemination recommendations ( WHO, 2014) and a Patient and Public Involvement (PPI) Ignite developed PPI Dissemination Planning tool. As a priority, we aim to disseminate the findings of the study to local, national and international audiences via relevant meetings, conferences and peer-reviewed academic journals. All outputs will be prepared using language that is appropriate for the target audience.

### Study status

The study is currently ongoing. The expected end date for the study is April 2023.

## Discussion

This protocol outlines the design of the study titled ‘ *Music and Movement for Health: Protocol for a pragmatic cluster-randomised feasibility pilot trial of an arts-based programme for the health and wellbeing of older adults*’. The aim of the *Music and Movement for Health* project is to assess the feasibility of a comprehensively designed complex arts-based programme that has the potential to target several important interrelated psychosocial and physical factors in older adults. The programme will be developed through a systematic multi-stage process to optimise effect, buy-in and participation. The overall aim is to evaluate the feasibility of a future definitive pragmatic, cluster-randomised, controlled trial to evaluate the effects of a tailored community-based arts-based health programme for older adults in a community setting in Ireland. The study also aims to examine if, how, and why the arts-based programme was successful at producing the desired outcomes. To the best of our knowledge, such a study has not been previously conducted in the Irish context. Given that a significant number of older adults are not meeting the recommended physical activity guidelines as well as the impact of the COVID-19 pandemic on levels of social isolation, loneliness and physical deconditioning, the assessment of a community-based arts programme is a priority for research as well as for the provision of services. This study would be a significant step in gauging the feasibility and helpfulness of a community arts-based health programme and would be of benefit to clinicians, service-planners and policymakers working in the field of older adult healthcare.

## Data availability

### Underlying data

No data is associated with this article.

### Extended data

Open Science Framework: Music and Movement for Health: Protocol for a pragmatic cluster-randomised feasibility pilot trial of an arts-based programme for the health and wellbeing of older adults. https://doi.org/10.17605/OSF.IO/32BTX ( Clifford, 2022).

This project contains the following extended data:

- Appendix 1: WHO registration data set
- Appendix 2: CONSORT 2010 Flow Diagram Movement and Music for Health
- Appendix 3: Participant Consent Form

### Reporting guidelines

Open Science Framework: SPIRIT checklist for ‘ *Music and Movement for Health*: Protocol for a pragmatic cluster-randomised feasibility pilot trial of an arts-based programme for the health and wellbeing of older adults’. https://doi.org/10.17605/OSF.IO/32BTX ( Clifford, 2022).

- Appendix 4: SPIRIT 2013 Checklist: Recommended items to address in a clinical trial protocol and related documents
- Appendix 5: CONSORT 2010 checklist of information to include when reporting a cluster-randomised trial
- Appendix 6: CONSORT 2010 checklist of information to include when reporting a pilot or feasibility trial

Data are available under the terms of the Creative Commons Zero "No rights reserved" data waiver (CC0 1.0 Public domain dedication).
