# Supplementary material for: Music and Movement for Health: Protocol for a pragmatic cluster-randomised feasibility pilot trial of an arts-based programme for the health and wellbeing of older adults
Source: HRB Open Res. 2023 Mar 29;5:42. Originally published 2022 May 26. [Version 2] doi: 10.12688/hrbopenres.13535.2 (PMC9843089; doi:10.12688/hrbopenres.13535.2)
Supplement: Peer review history

# Peer review: Reviewer response for version 2

**DOI:** 10.21956/hrbopenres.15003.r33590

**Published:** 2023-04-28

**Authors:** Aline Nogueira Haas

**Affiliations:** 1 School of Physical Education, Universidade Federal do Rio Grande do Sul, Porto Alegre, Brazil

I have no further comments to make.

Is the study design appropriate for the research question?

Yes

Is the rationale for, and objectives of, the study clearly described?

Yes

Are sufficient details of the methods provided to allow replication by others?

Yes

Are the datasets clearly presented in a useable and accessible format?

Yes

Reviewer Expertise:

Dance. Health. Parkinson's Disease.

I confirm that I have read this submission and believe that I have an appropriate level of expertise to confirm that it is of an acceptable scientific standard.

# Peer review: Reviewer response for version 1

**DOI:** 10.21956/hrbopenres.14772.r33269

**Published:** 2023-01-16

**Authors:** Aline Nogueira Haas

**Affiliations:** 1 School of Physical Education, Universidade Federal do Rio Grande do Sul, Porto Alegre, Brazil

This is a pragmatic cluster-randomised controlled feasibility trial that aims to the feasibility of the study design, its application to a music and movement for health programme and associated costs. The secondary aim is of the study is to obtain preliminary effect estimates of an ABHP on health and wellbeing in older adults.

 The Introduction is clear and theory based, showing the state of the art, and using current references. The methods are appropriate: the study design is clear; and the participants, instruments and outcomes are describe in details.

 Some information about the intervention was presented in the methods section. However, it will be useful to have more details about how and when the intervention will be administered. It is necessary to provide sufficient details that allow replication. Could the authors provide a table and/or video and/or figure as a supplementary material with more details about the intervention?

Is the study design appropriate for the research question?

Yes

Is the rationale for, and objectives of, the study clearly described?

Yes

Are sufficient details of the methods provided to allow replication by others?

Yes

Are the datasets clearly presented in a useable and accessible format?

Yes

Reviewer Expertise:

Dance. Health. Parkinson's Disease.

I confirm that I have read this submission and believe that I have an appropriate level of expertise to confirm that it is of an acceptable scientific standard.

# Peer review: Reviewer response for version 1

**DOI:** 10.21956/hrbopenres.14772.r32215

**Published:** 2022-07-05

**Authors:** Roger O'Sullivan, Laura McQuade

**Affiliations:** 1 Institute of Public Health in Ireland, Belfast, UK; 2 Bamford Centre for Mental Health and Well Being, Ulster University, Coleraine, UK

Thank you for the opportunity to review the protocol for this pragmatic cluster-randomised feasibility pilot trial investigating the impact of an arts-based intervention of music and movement on health and wellbeing in older adults.

 This feasibility pilot study by Clifford *et al*. is highly welcomed and can help make an important contribution to this developing and growing field. The aims of the study are well-defined and examine the:

1. Feasibility of the study design through a process evaluation (including an economic evaluation) and
2. Health outcomes associated with older adults’ participation in the 12-week arts-based intervention.

The protocol is clearly written with sufficient detail and addresses the key requirements for the standards of good practice for a pilot randomised control trial.

 A particular strength of the proposal is the spread of the disciplines involved and the potential for a pooled body of knowledge that can help increase our understanding in this area. Furthermore, the use of the expert workshops (health care practitioners, music therapists, dance teachers, and researchers) and Patient and Public Involvement which informs the study design and process will be insightful in providing practical user based informed experiences.

 The authors may wish to consider the following aspects:

- The protocol states that there is a screening process for inclusion and exclusion of participants into the study and some screening detail has been provided; however, it is not clear which standardised tools will be used to assess the criteria (e.g., levels of literacy).
- Linked to the above point and given the focus, further details on how to screen out any pre-existing cognitive, neurological conditions should be provided which will allow the team to control for any confounding factors in the analysis (e.g., using MMSE) and assist with replication. In addition, some outcome measures assessment could benefit from providing more specific detail, for example, falls history, falls log, and loneliness.
- A strength of the study is that a wide range of health outcomes will be assessed, however, consideration should also be given to participants' ability to fully complete all of the outcome assessments due to the length of time taken to complete them or physical endurance required to carry these out. It would be useful to provide some detail about how long this would take and an outline of how to reduce non-completion rates (e.g., regular breaks, etc).
- It would be helpful to set out in more detail the plans for recruitment on the qualitative aspect of the process evaluation, e.g., how many interviews or focus groups are planned, how many participants, and what sampling methods will be used to capture the range of perspectives.
- The literature from the field indicates that recruitment and attrition rates are two significant aspects that require careful consideration. The protocol acknowledges the issue of recruiting and sustaining male involvement, but further consideration and specificity could be given on the plans to achieve a gender balance and assess the strategy which will be undertaken as part of the study. It may be useful for the authors to consider the findings from work that focuses on men’s participation in an arts intervention (O’Donnell, 2021 ^1^ and O’Donnell *et al*., 2021 ^2^).
- Reference on page 3 within the protocol (IPH, 2021) should be amended to: McQuade L. and O’Sullivan R. Arts and Creativity in Later Life: Implications for Health and Wellbeing in Older Adults. A Systematic Evidence Review. Institute of Public Health. 2021 ^3^.
- With regard to the economic assessment of arts and creativity interventions, it may be useful for the authors to consult this piece of work by Crealey and O’Neill (2021) ^4^.

We wish the authors every success with this feasibility pilot trial study and look forward to reading the findings in the future.

Is the study design appropriate for the research question?

Yes

Is the rationale for, and objectives of, the study clearly described?

Yes

Are sufficient details of the methods provided to allow replication by others?

Partly

Are the datasets clearly presented in a useable and accessible format?

Not applicable

Reviewer Expertise:

Ageing, Public Health Research, Evaluation, Systematic Reviews; Arts and Creativity; Loneliness; Social Isolation; Physical Activity.

We confirm that we have read this submission and believe that we have an appropriate level of expertise to confirm that it is of an acceptable scientific standard.

## References

[1] : Development of a participatory arts intervention to promote mental health and wellbeing among men ‘at risk’ of suicide. *Queen’s University Belfast* .2021; Reference source

[2] : The acceptability, effectiveness and gender responsiveness of participatory arts interventions in promoting mental health and Wellbeing: a systematic review. *Arts Health* .14(2) : 10.1080/17533015.2021.1894463 186-203 10.1080/17533015.2021.1894463 33661710

[3] : Arts and Creativity in Later Life: Implications for Health and Wellbeing in Older Adults Systematic Evidence Review. *Institute of Public Health* .2021; Reference source

[4] : A review of economic evaluation methodologies for the assessment of arts and creativity interventions for improving health and wellbeing in older adults. *Institute of Public Health* .2021; Reference source 10.1186/s12889-023-17369-xPMC1071750338093290
